# *catena*-Poly[diaqua­tris(μ_3_-biphenyl-2,2-dicarboxyl­ato)disamarium(III)]

**DOI:** 10.1107/S1600536809046625

**Published:** 2009-11-11

**Authors:** Dan-Yi Wei, Yan-Guang Zhang, Mei-Li Wang, Zhen-Ke Zhu

**Affiliations:** a State Key Laboratory Base of Novel Functional Materials and Preparation Science, Faculty of Materials Science and Chemical Engineering, Ningbo University, Ningbo, Zhejiang 315211, People’s Republic of China

## Abstract

The title compound, [Sm_2_(C_14_H_8_O_4_)_3_(H_2_O)_2_]_*n*_, is composed of one-dimensional chains and is isostructural with previously reported compounds [Wang *et al.* (2003 ▶). *Eur. J. Inorg. Chem.* pp. 1355–1360]. The asymmetric unit contains two Sm atoms, each of which lies on a crystallographic twofold axis. Both crystallographically independent Sm atoms are coordinated by eight O atoms in a distorted dodeca­hedral arrangement. The polymeric chains run along [001]. Adjacent chains are connected through π–π inter­actions [centroid–centroid distance = 3.450 (2) Å], forming a two-dimensional supra­molecular network.

## Related literature

For background to the design and syntheses of lanthanide complexes and their potential applications as fluorescent probes, magnetic materials and catalysts, see: Barta *et al.* (2008 ▶); de Bettencourt-Dias *et al.* (2005 ▶), (2005); Chen *et al.* (2008 ▶); Fujita *et al.* (1994 ▶); Taniguchi & Takahei (1993 ▶). For the effect of the organic ligands on the structural framework of lanthanide complexes, see: Liu & Xu (2005 ▶); Wang *et al.* (2007 ▶); Yigit *et al.* (2006 ▶). For the use of multidentate *O*-donor ligands as organic spacers in the construction of these complexes, see: Lin *et al.* (2005 ▶); Zheng *et al.* (2008 ▶). For the coordination behaviour of 2,2′-biphenyl­dicarboxyl­ate, see: Thirumurugan *et al.* (2003 ▶); Xu *et al.* (2006 ▶); Rui *et al.* (2007 ▶).
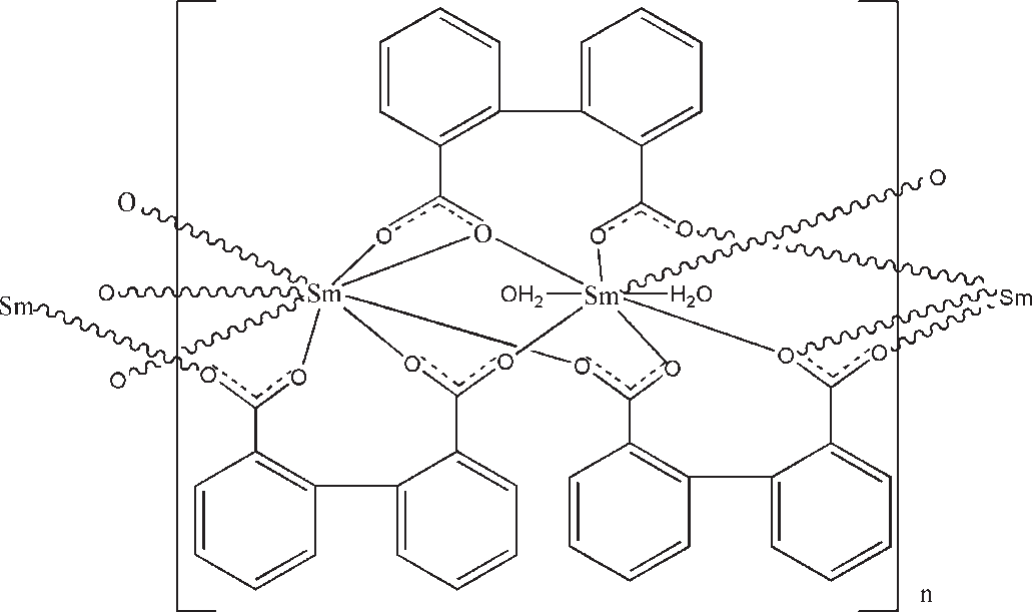


## Experimental

### 

#### Crystal data

[Sm_2_(C_14_H_8_O_4_)_3_(H_2_O)_2_]
*M* *_r_* = 1057.34
Monoclinic, 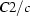

*a* = 20.776 (4) Å
*b* = 21.441 (4) Å
*c* = 8.2660 (17) Å
β = 103.94 (3)°
*V* = 3573.7 (13) Å^3^
*Z* = 4
Mo *K*α radiation
μ = 3.33 mm^−1^
*T* = 293 K
0.39 × 0.34 × 0.27 mm

#### Data collection

Rigaku R-AXIS RAPID diffractometer
Absorption correction: multi-scan (*ABSCOR*; Higashi, 1995 ▶) *T*_min_ = 0.261, *T*_max_ = 0.409
15001 measured reflections
3962 independent reflections
3238 reflections with *I* > 2σ(*I*)
*R*_int_ = 0.067

#### Refinement

*R*[*F* ^2^ > 2σ(*F* ^2^)] = 0.066
*wR*(*F* ^2^) = 0.130
*S* = 1.18
3962 reflections
263 parameters
H-atom parameters constrained
Δρ_max_ = 2.31 e Å^−3^
Δρ_min_ = −1.91 e Å^−3^

### 

Data collection: *RAPID-AUTO* (Rigaku, 1998 ▶); cell refinement: *RAPID-AUTO*; data reduction: *CrystalStructure* (Rigaku/MSC, 2002 ▶); program(s) used to solve structure: *SHELXS97* (Sheldrick, 2008 ▶); program(s) used to refine structure: *SHELXL97* (Sheldrick, 2008 ▶); molecular graphics: *ORTEPII* (Johnson, 1976 ▶); software used to prepare material for publication: *SHELXL97*.

## Supplementary Material

Crystal structure: contains datablocks global, I. DOI: 10.1107/S1600536809046625/jh2091sup1.cif

Structure factors: contains datablocks I. DOI: 10.1107/S1600536809046625/jh2091Isup2.hkl

Additional supplementary materials:  crystallographic information; 3D view; checkCIF report

## Figures and Tables

**Fig. 1.**
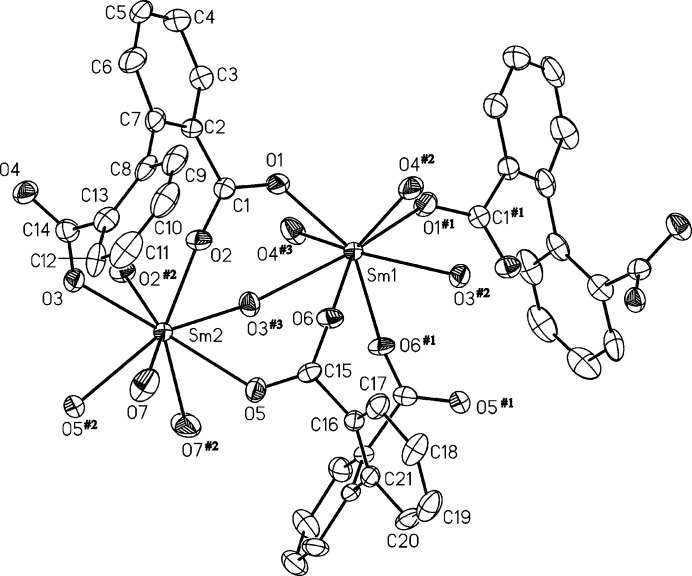
ORTEP view of complex molecule of (I). Displacement ellipsoids are drawn at the 45% probability level. (#1 = -x, y, 3/2 - z; #2 = x, y, z + 1; #3 = -x, y, 1/2 - z.)

**Fig. 2.**
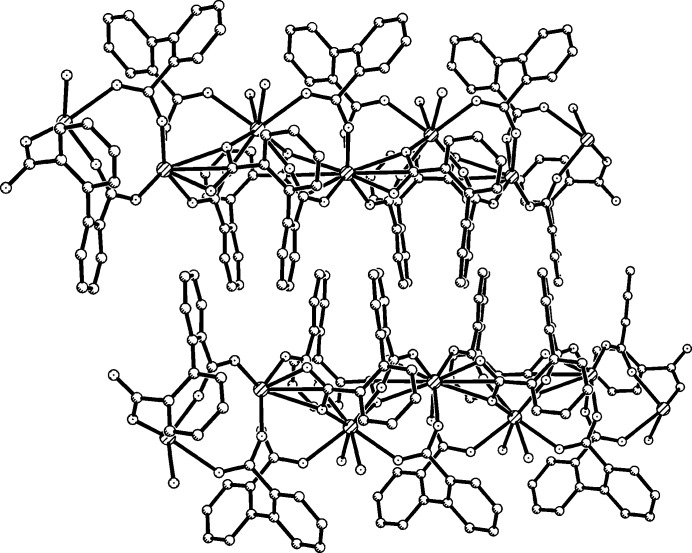
two-dimensional supramolecular layer in (I).

## supplementary crystallographic information

### Comment

Design and syntheses of lanthanide complexes are of great interest due to their various topological networks and potential applications in fluorescent probes, magnetic materials, catalysts (Barta *et al.*, 2008; Bettencout-de Dias, 2005; Chen *et al.*, 2008; Fujita *et al.*, 1994; Taniguchi & Takahei, 1993). As reported in literature, the geometries and properties of organic ligands have great effect on structural framework of lanthanide complexes. So much effort has been devoted to modify the building blocks to control the products by selection of appropriate organic ligands (Liu & Xu, 2005; Wang *et al.*, 2007; Yigit *et al.*, 2006). Multidentae O donor ligands have been employed extensively as organic spacers in the construction of these complexes, such as α,ω-dicarboxylate and 1,3,5-benzenetricarboxylate (Lin *et al.*, 2005; Zheng *et al.*, 2008). Recently, research suggests that 2,2'-biphenyldicarboxylate (dpdc) possesses intriguing coordination behaviors to afford new coordination polymers (Thirumurugan *et al.*, 2003; Xu, *et al.*, 2006; Rui, *et al.*, 2007). In this articles, we will report a new coordination polymers [Sm_2_(C_14_H_8_O_4_)_3_(H_2_O)_2_]_n_.

The crystal structure of the title compound (I) consists of one-dimensional chains of [Sm_2_(C_14_H_8_O_4_)_3_(H_2_O)_2_]_n_. (Fig 1). The asymmetric unit consists of two Sm atoms, each of which lies on the crystallographic twofold axis. Both crystallographically independent Sm atoms are coordinated to eight oxygen atoms and have a distorted dodecahedral arrangement. The Sm(1)—O (carboxylate) distances fall in the range 2.320 (5)–2.615 (5) Å, and the O—Sm(1)—O bond angles are in the range 25.5 (2)–153.5 (2)°. While the Sm(2)—O (carboxylate) bond lengths vary from 2.301 (5) Å to 2.480 (5))Å, and the Sm(2)—O (aqua) distances are both 2.540 (6) Å. The O—Sm(2)—O bond angles range from 66.8 (2)–147.2 (2)°. The coordination environments of two Sm atoms are different. The Sm(1) is coordinated to one tetradentate dpdc ligand and four pentadentate dpdc ligands, however, the Sm(2) is bonded to two tetradentate dpdc ligands, two pentadentate dpdc ligands, and two coordinated water molecules. The Sm atoms are bridged by the two types dpdc ligands to afford one-dimensional infinite polymeric chain which run along the [001] direction. As reported in documents, the one-dimensional chain looks like a pinwheel, the Sm atoms are at the center of pinwheel. The parallel phenyl rings of adjacent chains are interdigitaed. The two-dimensional supramolecule networks are formed by π-π interactions between these phenyl rings.

### Experimental

A mixture of Sm(NO_3_)_3_.6H_2_O (0.222 g, 0.5 mmol) and 2,2'-diphenldicarboxylic acid (0.126 g, 0.5 mmol), H_2_O (5 ml), and H_2_C_2_O_4_ (0.080 g, 1 mmol), NaOH (0.040 g, 1 mmol) was sealed in a 25-ml stainless-steel reactor with Teflon liner, heated to 180°C for 4 days, and then cooled to room temperature. The products were filtered and colorless block crystals are obtained.

### Refinement

H atoms bonded to C atoms were placed in geometrically calulated positons and refined using a riding moldel, with *U*_iso_(H) = 1.2Ueq(C). Water H atoms were found in difference Fourier synthesis and refined with th O—H distances fixed as initially found, with *U*_iso_(H) = 1.2Ueq(O).

### Crystal data

**Table:**

[Sm_2_(C_14_H_8_O_4_)_3_(H_2_O)_2_]	*F*(000) = 2064
*M**_r_* = 1057.34	*D*_x_ = 1.965 Mg m^−^^3^
Monoclinic, *C*2/*c*	Mo *K*α radiation, λ = 0.71073 Å
Hall symbol: -C 2yc	Cell parameters from 12337 reflections
*a* = 20.776 (4) Å	θ = 3.0–27.7°
*b* = 21.441 (4) Å	µ = 3.33 mm^−^^1^
*c* = 8.2660 (17) Å	*T* = 293 K
β = 103.94 (3)°	Block, colorless
*V* = 3573.7 (13) Å^3^	0.39 × 0.34 × 0.27 mm
*Z* = 4	

### Data collection

**Table:**

Rigaku R-AXIS RAPID diffractometer	3962 independent reflections
Radiation source: fine-focus sealed tube	3238 reflections with *I* > 2σ(*I*)
graphite	*R*_int_ = 0.067
Detector resolution: 0 pixels mm^-1^	θ_max_ = 27.5°, θ_min_ = 3.0°
ω scans	*h* = −26→26
Absorption correction: multi-scan (*ABSCOR*; Higashi, 1995)	*k* = −27→27
*T*_min_ = 0.261, *T*_max_ = 0.409	*l* = −10→10
15001 measured reflections	

### Refinement

**Table:**

Refinement on *F*^2^	Primary atom site location: structure-invariant direct methods
Least-squares matrix: full	Secondary atom site location: difference Fourier map
*R*[*F*^2^ > 2σ(*F*^2^)] = 0.066	Hydrogen site location: inferred from neighbouring sites
*wR*(*F*^2^) = 0.130	H-atom parameters constrained
*S* = 1.18	*w* = 1/[σ^2^(*F*_o_^2^) + 89.0505*P*] where *P* = (*F*_o_^2^ + 2*F*_c_^2^)/3
3962 reflections	(Δ/σ)_max_ = 0.005
263 parameters	Δρ_max_ = 2.31 e Å^−^^3^
0 restraints	Δρ_min_ = −1.91 e Å^−^^3^

### Special details

**Table:**

Geometry. All esds (except the esd in the dihedral angle between two l.s. planes) are estimated using the full covariance matrix. The cell esds are taken into account individually in the estimation of esds in distances, angles and torsion angles; correlations between esds in cell parameters are only used when they are defined by crystal symmetry. An approximate (isotropic) treatment of cell esds is used for estimating esds involving l.s. planes.
Refinement. Refinement of F^2^ against ALL reflections. The weighted R-factor wR and goodness of fit S are based on F^2^, conventional R-factors R are based on F, with F set to zero for negative F^2^. The threshold expression of F^2^ > 2sigma(F^2^) is used only for calculating R-factors(gt) etc. and is not relevant to the choice of reflections for refinement. R-factors based on F^2^ are statistically about twice as large as those based on F, and R- factors based on ALL data will be even larger.

### Fractional atomic coordinates and isotropic or equivalent isotropic displacement parameters (Å^2^)

**Table:**

	*x*	*y*	*z*	*U*_iso_*/*U*_eq_	
Sm1	0.0000	0.20641 (2)	0.7500	0.01913 (15)	
Sm2	0.0000	0.28693 (2)	0.2500	0.01920 (15)	
O1	0.0507 (3)	0.1479 (3)	0.5795 (7)	0.0347 (14)	
O2	0.0696 (3)	0.2088 (2)	0.3778 (7)	0.0270 (12)	
O3	0.0595 (3)	0.2463 (3)	0.0471 (7)	0.0272 (12)	
O4	0.0957 (3)	0.1637 (3)	−0.0570 (7)	0.0371 (15)	
O5	0.0340 (3)	0.3549 (3)	0.4988 (7)	0.0314 (14)	
O6	0.0664 (3)	0.2896 (2)	0.7115 (7)	0.0253 (12)	
C1	0.0717 (4)	0.1566 (4)	0.4500 (9)	0.0219 (15)	
C2	0.0997 (4)	0.1010 (4)	0.3809 (10)	0.0261 (17)	
C3	0.0705 (5)	0.0434 (4)	0.3985 (11)	0.038 (2)	
H3A	0.0340	0.0418	0.4451	0.045*	
C4	0.0948 (6)	−0.0106 (4)	0.3481 (12)	0.047 (3)	
H4A	0.0750	−0.0487	0.3608	0.056*	
C5	0.1484 (6)	−0.0084 (5)	0.2790 (11)	0.051 (3)	
H5A	0.1650	−0.0448	0.2430	0.062*	
C6	0.1774 (5)	0.0479 (5)	0.2632 (12)	0.043 (2)	
H6A	0.2147	0.0484	0.2194	0.051*	
C7	0.1542 (4)	0.1042 (4)	0.3090 (10)	0.0303 (19)	
C8	0.1910 (4)	0.1616 (4)	0.2945 (10)	0.0290 (18)	
C9	0.2548 (5)	0.1680 (5)	0.3969 (12)	0.043 (2)	
H9A	0.2710	0.1377	0.4768	0.051*	
C10	0.2938 (5)	0.2178 (5)	0.3824 (14)	0.049 (3)	
H10A	0.3359	0.2209	0.4529	0.059*	
C11	0.2722 (5)	0.2637 (6)	0.2657 (14)	0.047 (3)	
H11A	0.2996	0.2971	0.2555	0.057*	
C12	0.2084 (5)	0.2593 (5)	0.1633 (10)	0.037 (2)	
H12A	0.1926	0.2902	0.0849	0.045*	
C13	0.1687 (4)	0.2091 (4)	0.1778 (11)	0.0307 (19)	
C14	0.1034 (4)	0.2052 (4)	0.0510 (10)	0.0277 (17)	
C15	0.0564 (4)	0.3429 (3)	0.6499 (10)	0.0226 (16)	
C16	0.0779 (4)	0.3973 (3)	0.7700 (9)	0.0246 (17)	
C17	0.1438 (4)	0.3978 (4)	0.8573 (10)	0.0294 (18)	
H17A	0.1715	0.3649	0.8458	0.035*	
C18	0.1689 (5)	0.4476 (5)	0.9620 (12)	0.040 (2)	
H18A	0.2131	0.4478	1.0206	0.048*	
C19	0.1282 (5)	0.4962 (4)	0.9784 (11)	0.041 (2)	
H19A	0.1452	0.5300	1.0458	0.049*	
C20	0.0622 (5)	0.4954 (4)	0.8956 (11)	0.034 (2)	
H20A	0.0348	0.5282	0.9097	0.041*	
C21	0.0358 (5)	0.4454 (3)	0.7900 (10)	0.0278 (18)	
O7	0.0919 (4)	0.3610 (3)	0.2356 (8)	0.0474 (18)	
H7B	0.1218	0.3708	0.3270	0.050*	
H7A	0.1034	0.3561	0.1493	0.075*	

### Atomic displacement parameters (Å^2^)

**Table:**

	*U*^11^	*U*^22^	*U*^33^	*U*^12^	*U*^13^	*U*^23^
Sm1	0.0230 (3)	0.0164 (3)	0.0182 (3)	0.000	0.0054 (2)	0.000
Sm2	0.0226 (3)	0.0174 (3)	0.0179 (3)	0.000	0.0056 (2)	0.000
O1	0.054 (4)	0.025 (3)	0.033 (3)	0.009 (3)	0.025 (3)	0.005 (2)
O2	0.025 (3)	0.023 (3)	0.033 (3)	0.005 (2)	0.009 (2)	0.002 (2)
O3	0.029 (3)	0.031 (3)	0.023 (3)	0.005 (2)	0.009 (2)	−0.001 (2)
O4	0.041 (4)	0.032 (3)	0.032 (3)	0.013 (3)	−0.004 (3)	−0.007 (2)
O5	0.047 (4)	0.027 (3)	0.022 (3)	−0.007 (3)	0.012 (3)	−0.003 (2)
O6	0.022 (3)	0.019 (3)	0.037 (3)	−0.004 (2)	0.011 (2)	0.001 (2)
C1	0.017 (4)	0.025 (4)	0.024 (4)	0.004 (3)	0.007 (3)	−0.002 (3)
C2	0.031 (4)	0.023 (4)	0.023 (4)	0.012 (3)	0.004 (3)	0.000 (3)
C3	0.051 (6)	0.028 (4)	0.034 (5)	0.002 (4)	0.010 (4)	0.000 (3)
C4	0.077 (8)	0.024 (5)	0.036 (6)	0.004 (5)	0.007 (5)	0.000 (4)
C5	0.090 (9)	0.038 (5)	0.018 (5)	0.029 (6)	−0.001 (5)	−0.008 (4)
C6	0.042 (6)	0.047 (6)	0.037 (5)	0.025 (5)	0.004 (4)	−0.009 (4)
C7	0.033 (5)	0.035 (5)	0.018 (4)	0.014 (4)	−0.002 (3)	−0.004 (3)
C8	0.021 (4)	0.039 (5)	0.029 (4)	0.010 (3)	0.009 (3)	−0.010 (3)
C9	0.031 (5)	0.057 (6)	0.038 (5)	0.022 (5)	0.005 (4)	−0.015 (4)
C10	0.022 (5)	0.069 (7)	0.054 (7)	0.004 (5)	0.006 (4)	−0.028 (5)
C11	0.032 (5)	0.058 (7)	0.053 (7)	−0.015 (5)	0.015 (5)	−0.023 (5)
C12	0.043 (6)	0.061 (6)	0.010 (4)	−0.005 (5)	0.012 (4)	−0.007 (3)
C13	0.019 (4)	0.037 (5)	0.036 (5)	0.005 (3)	0.006 (3)	−0.008 (3)
C14	0.030 (4)	0.027 (4)	0.026 (4)	−0.004 (3)	0.006 (3)	0.003 (3)
C15	0.018 (4)	0.021 (4)	0.032 (4)	0.000 (3)	0.013 (3)	−0.005 (3)
C16	0.034 (5)	0.019 (4)	0.022 (4)	−0.006 (3)	0.010 (3)	−0.002 (3)
C17	0.034 (5)	0.033 (4)	0.026 (4)	−0.001 (4)	0.017 (4)	−0.004 (3)
C18	0.035 (5)	0.048 (6)	0.037 (5)	−0.009 (4)	0.011 (4)	−0.016 (4)
C19	0.055 (6)	0.040 (5)	0.031 (5)	−0.017 (5)	0.016 (4)	−0.017 (4)
C20	0.049 (6)	0.019 (4)	0.043 (5)	−0.006 (4)	0.026 (4)	−0.009 (3)
C21	0.047 (5)	0.015 (3)	0.027 (4)	−0.007 (3)	0.019 (4)	−0.001 (3)
O7	0.053 (4)	0.052 (4)	0.043 (4)	−0.026 (4)	0.023 (3)	−0.018 (3)

### Geometric parameters (Å, °)

**Table:**

Sm1—O1^i^	2.321 (6)	C4—H4A	0.9300
Sm1—O1	2.321 (6)	C5—C6	1.370 (15)
Sm1—O6	2.322 (5)	C5—H5A	0.9300
Sm1—O6^i^	2.322 (5)	C6—C7	1.386 (11)
Sm1—O4^ii^	2.410 (6)	C6—H6A	0.9300
Sm1—O4^iii^	2.410 (6)	C7—C8	1.469 (13)
Sm1—O3^iii^	2.613 (5)	C8—C9	1.397 (12)
Sm1—O3^ii^	2.613 (5)	C8—C13	1.403 (12)
Sm1—C14^iii^	2.869 (8)	C9—C10	1.362 (15)
Sm1—C14^ii^	2.869 (8)	C9—H9A	0.9300
Sm2—O2^ii^	2.298 (5)	C10—C11	1.375 (16)
Sm2—O2	2.298 (5)	C10—H10A	0.9300
Sm2—O3^ii^	2.469 (6)	C11—C12	1.394 (13)
Sm2—O3	2.469 (6)	C11—H11A	0.9300
Sm2—O5	2.480 (5)	C12—C13	1.377 (13)
Sm2—O5^ii^	2.480 (5)	C12—H12A	0.9300
Sm2—O7	2.509 (6)	C13—C14	1.503 (11)
Sm2—O7^ii^	2.509 (6)	C14—Sm1^iv^	2.869 (8)
O1—C1	1.264 (9)	C15—C16	1.527 (10)
O2—C1	1.263 (9)	C16—C17	1.387 (12)
O3—C14	1.264 (10)	C16—C21	1.388 (11)
O3—Sm1^iv^	2.613 (5)	C17—C18	1.394 (12)
O4—C14	1.244 (10)	C17—H17A	0.9300
O4—Sm1^iv^	2.410 (6)	C18—C19	1.369 (14)
O5—C15	1.250 (10)	C18—H18A	0.9300
O6—C15	1.248 (9)	C19—C20	1.376 (14)
C1—C2	1.498 (10)	C19—H19A	0.9300
C2—C3	1.399 (12)	C20—C21	1.407 (11)
C2—C7	1.402 (12)	C20—H20A	0.9300
C3—C4	1.369 (13)	C21—C21^i^	1.477 (18)
C3—H3A	0.9300	O7—H7B	0.8798
C4—C5	1.370 (16)	O7—H7A	0.8122
			
O1^i^—Sm1—O1	114.5 (3)	C14—O3—Sm2	134.8 (5)
O1^i^—Sm1—O6	150.4 (2)	C14—O3—Sm1^iv^	88.3 (5)
O1—Sm1—O6	87.7 (2)	Sm2—O3—Sm1^iv^	123.6 (2)
O1^i^—Sm1—O6^i^	87.7 (2)	C14—O4—Sm1^iv^	98.4 (5)
O1—Sm1—O6^i^	150.4 (2)	C15—O5—Sm2	132.1 (5)
O6—Sm1—O6^i^	79.7 (3)	C15—O6—Sm1	135.5 (5)
O1^i^—Sm1—O4^ii^	76.9 (2)	O2—C1—O1	123.5 (7)
O1—Sm1—O4^ii^	79.4 (2)	O2—C1—C2	119.8 (7)
O6—Sm1—O4^ii^	128.6 (2)	O1—C1—C2	116.7 (7)
O6^i^—Sm1—O4^ii^	87.7 (2)	C3—C2—C7	120.1 (8)
O1^i^—Sm1—O4^iii^	79.4 (2)	C3—C2—C1	116.4 (8)
O1—Sm1—O4^iii^	76.9 (2)	C7—C2—C1	123.4 (7)
O6—Sm1—O4^iii^	87.7 (2)	C4—C3—C2	121.0 (10)
O6^i^—Sm1—O4^iii^	128.6 (2)	C4—C3—H3A	119.5
O4^ii^—Sm1—O4^iii^	135.3 (3)	C2—C3—H3A	119.5
O1^i^—Sm1—O3^iii^	77.7 (2)	C3—C4—C5	119.7 (10)
O1—Sm1—O3^iii^	124.5 (2)	C3—C4—H4A	120.2
O6—Sm1—O3^iii^	73.45 (19)	C5—C4—H4A	120.2
O6^i^—Sm1—O3^iii^	77.39 (19)	C6—C5—C4	119.4 (9)
O4^ii^—Sm1—O3^iii^	151.0 (2)	C6—C5—H5A	120.3
O4^iii^—Sm1—O3^iii^	51.29 (18)	C4—C5—H5A	120.3
O1^i^—Sm1—O3^ii^	124.5 (2)	C5—C6—C7	123.5 (11)
O1—Sm1—O3^ii^	77.7 (2)	C5—C6—H6A	118.2
O6—Sm1—O3^ii^	77.39 (19)	C7—C6—H6A	118.2
O6^i^—Sm1—O3^ii^	73.45 (19)	C6—C7—C2	116.3 (9)
O4^ii^—Sm1—O3^ii^	51.29 (18)	C6—C7—C8	119.1 (9)
O4^iii^—Sm1—O3^ii^	151.0 (2)	C2—C7—C8	124.4 (7)
O3^iii^—Sm1—O3^ii^	141.8 (2)	C9—C8—C13	117.0 (9)
O1^i^—Sm1—C14^iii^	79.8 (2)	C9—C8—C7	118.0 (8)
O1—Sm1—C14^iii^	99.6 (2)	C13—C8—C7	124.9 (7)
O6—Sm1—C14^iii^	77.3 (2)	C10—C9—C8	121.4 (10)
O6^i^—Sm1—C14^iii^	103.5 (2)	C10—C9—H9A	119.3
O4^ii^—Sm1—C14^iii^	153.7 (2)	C8—C9—H9A	119.3
O4^iii^—Sm1—C14^iii^	25.4 (2)	C9—C10—C11	121.5 (9)
O3^iii^—Sm1—C14^iii^	26.1 (2)	C9—C10—H10A	119.3
O3^ii^—Sm1—C14^iii^	154.7 (2)	C11—C10—H10A	119.3
O1^i^—Sm1—C14^ii^	99.6 (2)	C10—C11—C12	118.6 (10)
O1—Sm1—C14^ii^	79.8 (2)	C10—C11—H11A	120.7
O6—Sm1—C14^ii^	103.5 (2)	C12—C11—H11A	120.7
O6^i^—Sm1—C14^ii^	77.3 (2)	C13—C12—C11	120.2 (10)
O4^ii^—Sm1—C14^ii^	25.4 (2)	C13—C12—H12A	119.9
O4^iii^—Sm1—C14^ii^	153.7 (2)	C11—C12—H12A	119.9
O3^iii^—Sm1—C14^ii^	154.7 (2)	C12—C13—C8	121.4 (8)
O3^ii^—Sm1—C14^ii^	26.1 (2)	C12—C13—C14	116.2 (8)
C14^iii^—Sm1—C14^ii^	179.0 (3)	C8—C13—C14	122.1 (8)
O2^ii^—Sm2—O2	86.4 (3)	O4—C14—O3	120.9 (8)
O2^ii^—Sm2—O3^ii^	72.10 (19)	O4—C14—C13	118.7 (8)
O2—Sm2—O3^ii^	78.1 (2)	O3—C14—C13	120.2 (7)
O2^ii^—Sm2—O3	78.1 (2)	O4—C14—Sm1^iv^	56.2 (4)
O2—Sm2—O3	72.10 (19)	O3—C14—Sm1^iv^	65.5 (4)
O3^ii^—Sm2—O3	138.7 (3)	C13—C14—Sm1^iv^	164.9 (6)
O2^ii^—Sm2—O5	146.3 (2)	O6—C15—O5	125.6 (7)
O2—Sm2—O5	91.4 (2)	O6—C15—C16	116.1 (7)
O3^ii^—Sm2—O5	74.51 (19)	O5—C15—C16	118.2 (7)
O3—Sm2—O5	132.9 (2)	C17—C16—C21	120.2 (7)
O2^ii^—Sm2—O5^ii^	91.4 (2)	C17—C16—C15	116.3 (7)
O2—Sm2—O5^ii^	146.3 (2)	C21—C16—C15	123.5 (7)
O3^ii^—Sm2—O5^ii^	132.9 (2)	C16—C17—C18	120.3 (8)
O3—Sm2—O5^ii^	74.51 (19)	C16—C17—H17A	119.9
O5—Sm2—O5^ii^	108.0 (3)	C18—C17—H17A	119.9
O2^ii^—Sm2—O7	147.4 (2)	C19—C18—C17	119.8 (9)
O2—Sm2—O7	94.7 (2)	C19—C18—H18A	120.1
O3^ii^—Sm2—O7	140.0 (2)	C17—C18—H18A	120.1
O3—Sm2—O7	71.3 (2)	C18—C19—C20	120.4 (8)
O5—Sm2—O7	66.4 (2)	C18—C19—H19A	119.8
O5^ii^—Sm2—O7	69.9 (2)	C20—C19—H19A	119.8
O2^ii^—Sm2—O7^ii^	94.7 (2)	C19—C20—C21	120.7 (9)
O2—Sm2—O7^ii^	147.4 (2)	C19—C20—H20A	119.6
O3^ii^—Sm2—O7^ii^	71.3 (2)	C21—C20—H20A	119.6
O3—Sm2—O7^ii^	140.0 (2)	C16—C21—C20	118.6 (8)
O5—Sm2—O7^ii^	69.9 (2)	C16—C21—C21^i^	122.8 (6)
O5^ii^—Sm2—O7^ii^	66.4 (2)	C20—C21—C21^i^	118.5 (7)
O7—Sm2—O7^ii^	101.4 (4)	Sm2—O7—H7B	119.9
C1—O1—Sm1	137.2 (5)	Sm2—O7—H7A	110.3
C1—O2—Sm2	144.0 (5)	H7B—O7—H7A	119.3

Symmetry codes: (i) −*x*, *y*, −*z*+3/2; (ii) −*x*, *y*, −*z*+1/2; (iii) *x*, *y*, *z*+1; (iv) *x*, *y*, *z*−1.
